# Enhancing bioactivity, physicochemical, and pharmacokinetic properties of a nano-sized, anti-VEGFR2 Adnectin, through PASylation technology

**DOI:** 10.1038/s41598-019-39776-0

**Published:** 2019-02-27

**Authors:** Safieh Aghaabdollahian, Reza Ahangari Cohan, Dariush Norouzian, Fatemeh Davami, Mohammad Reza Asadi Karam, Fatemeh Torkashvand, Golnaz Vaseghi, Reza Moazzami, Sakineh Latif Dizaji

**Affiliations:** 1 Department of Nanobiotechnology, New Technologies Research Group, Pasteur Institute of Iran, Tehran, Iran; 2 Biotechnology Research Center, Pasteur Institute of Iran, Tehran, Iran; 3 Department of Molecular Biology, Pasteur Institute of Iran, Tehran, Iran; 4 Department of Biotechnology, Pasteur Institute of Iran, Tehran, Iran; 5 Isfahan Cardiovascular research center, Department of Pharmacology, Isfahan, Iran

## Abstract

The crucial role of VEGF receptor 2 (VEGFR2) signaling in the angiogenesis and metastasis of solid tumors has prompted the development of inhibitors with minimal bystander effects. Recently, Adnectin C has attracted attention for cancer treatment. To overcome the problematic properties of Adnectin, a novel form of Adnectin C has been designed by its fusion to a biodegradable polymeric peptide containing Pro/Ala/Ser (PAS) repetitive residues. *E. coli*-expressed recombinant fused and unfused proteins were compared in terms of bioactivity, physicochemical, and pharmacokinetic properties using standard methods. Dynamic light scattering (DLS) analysis of PASylated adnectin C revealed an approximate 2-fold increase in particle size with a slight change in the net charge. Additionally, fusion of the PAS sequence improved its stability against the growth of thermo-induced aggregated forms. The high receptor-binding and improved binding kinetic parameters of PASylated Adnectin C was confirmed by ELISA and surface plasmon resonance assays, respectively. Pharmacokinetic studies showed a noticeable increase in the terminal half-life of Adnectin C-PAS#1(200) by a factor of 4.57 after single dose by intravenous injection into female BALB/c mice. The results suggest that PASylation could offer a superior delivery strategy for developing Adnectin-derived drugs with improved patient compliance.

## Introduction

Despite the advances in medical care, cancer remains a major cause of death worldwide. A critical process in cancer development is angiogenesis, in which new blood vessels are formed by the over-reaction of the key mediators, VEGF-A and VEGFR2^1,2^. Because VEGFR2 signaling is mainly responsible for the pro-angiogenic effect of VEGF-A^1^, a concerted effort has been made to block VEGF-A signaling through the use of monoclonal antibodies (bevacizumab) and tyrosine kinase inhibitors (such as sunitinib and sorafenib) in cancer therapy^3^. However, anti-VEGF-A monoclonal antibodies cannot completely block VEGFR2 activation because other VEGF family members (VEGF-C and VEGF-D) can simulate VEGFR2 signaling and because of the lackluster specificity of tyrosine kinase inhibitors that can result in severe adverse effects^4^.

Next-generation immunotherapeutics have focused on bio-compounds which can effectively and specifically inhibit angiogenesis and cover the bystander effects of available drugs. Adnectin C, a new bioactive compound developed by mRNA display technology, blocks the VEGFR2 signaling pathway. Its high specificity for VEGFR2 bypasses the side effects of existing drugs^4–6^. Adnectin C is a structural derivation of the tenth domain of fibronectin type III (10^th^Fn3), which belongs to a novel binding protein superfamily called protein scaffolds. Adnectin has 94 residues which embrace a β-sandwich fold with seven strands and two/three loops without disulfide bonds. Like the complementary determining regions of an antibody, these flexible loops bind to the target protein and are subject to mutation to develop artificial binding proteins. In comparison with mAbs, Adnectins exhibit a better tissue penetration rate and higher affinity. Because of their inherent nature, Adnectins have high specificity and thermostability (Tm > 80 °C), low immunogenicity and are easily expressed as recombinant proteins in the bacterial hosts^7–10^. Nonetheless, their short *in vivo* half-life restricts the wide use of Adnectins in practice.

Adnexus (Bristol-Myers Squibb) has recently developed a polyethylene glycol (PEG) attached form of the protein CT-322 which has undergone phase II clinical trials^4^. The drawbacks of PEGylation technology including the toxic accumulation of the drug in the kidney, protein inactivation upon coupling with the polymer, immunogenicity, heterogeneity of PEGylated drugs, low yield of conjugation and issues related to downstream processing. These, as well as the cost, have motivated researchers to shift to recombinant-based approaches for *in vivo* half-life extension^11–13^. Genetic fusion of biodrugs to homo-amino acid polymers (HAP)^14^ or XTEN^15^ and polysialylation (PSA)^16,17^ are examples of recombinant-based approaches to address this shortcoming by increasing the size and hydrodynamic volume of biomolecules. HAPylation exhibits low hydrophilicity, moreover, long protein polymers are necessary to produce a sensible effect on the elongation of circulation time. PSA is a less advanced technology and requires precise homogeneic control of the product^17,18^. Furthermore, in comparison with the net charge of the PAS sequence, the negative charge of the XTEN peptide leads to repulsive interaction with negatively charged cell surfaces and the extracellular matrix and subsequent inappropriate distribution^19,20^.

PASylation, a promising biological substitute for PEGylation, is a flexible repetitive hydrophilic sequence of proline, alanine and serine amino acids 100–600 residues in length that are fused to the N- and/or C-terminus of the protein of interest. It prolongs the blood circulation time by a remarkable amount in the hydrodynamic volume of the macromolecule^21^. This technology offers the benefits of PEGylation without a change in biological activity or affinity for the target protein. It facilitates the production of biopharmaceuticals, because no *in vitro* coupling steps are required.

Although PASylated bio-compounds are resistant to serum proteases, they can efficiently be degraded by kidney enzymes, so no tubular accumulation or vacuolation has been seen for *in vivo* assays. There is no change in the isoelectric point (pI) of PASylated biocompounds owing to the fact that PAS polymer is composed of uncharged residues^21–23^. PASylation has been shown to improve the solubility, stability and biological activity of its fusion partner^24^. Studies on PASylated proteins of various lengths and sequences reveal that the *in vivo* residence time is strongly correlated with the increase of PAS sequence length. However, to select a suitable PAS sequence length for anticancer biomolecules, the tumor tissue penetration rate of the fused proteins should be considered in the pharmaceutical design^22^. Recent studies on the development of PASylated biodrugs like erythropoietin^25^, IFN-β1b^26^, type I interferon superagonist^27^, hGH, leptin^13^, coversin^28^, αHER2^29,30^ and αCD20 Fab fragments^23^ have shown an enhanced pharmacokinetic profile through reduction of renal clearance following increased size/hydrodynamic volume of the fusion protein. PASylation has a positive effect on solubility, and biological activity of IFN-β1b, furthermore, has enhanced tumor uptake of αHER2. PASylation has improved agonistic or antagonistic activity of leptin, and enhanced anti-hemolytic activity of coversin, *in Vitro*^13^.

The aim of the current study was to evaluate the effect of a PAS sequence with a length of 200 residues attached to Adnectin C on physicochemical and biological properties, as well as pharmacokinetic parameters of the protein. For this purpose, *E. coli*-expressed recombinant fused and unfused proteins were compared in terms of particle size, zeta potential, charge, intact mass, thermo-gravimetry, receptor binding, kinetic parameters, cell toxicity, anti-proliferative activity, cell migration activity and pharmacokinetic profile.

## Results

### Expression and identification

**Figure 1:**
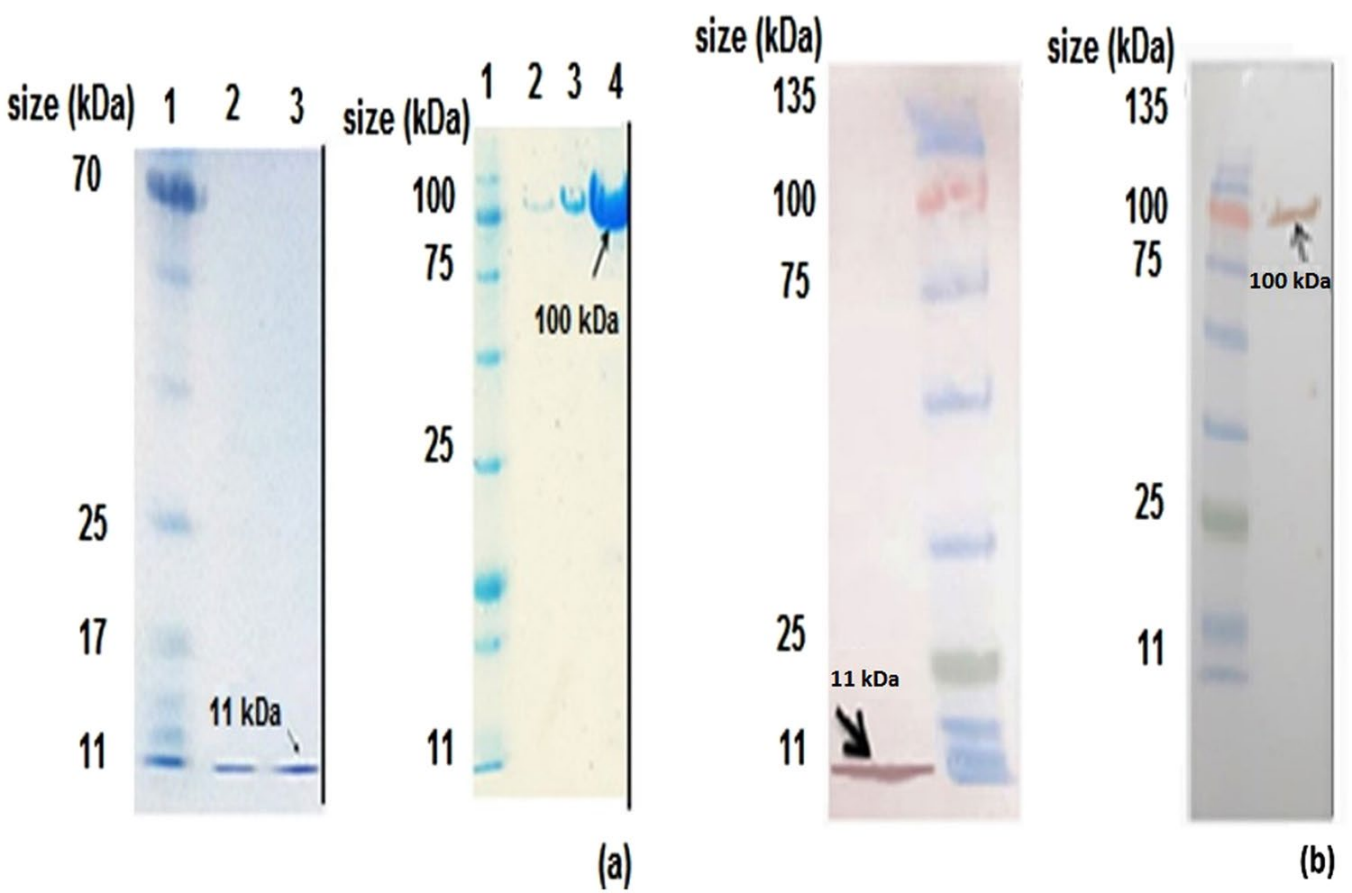
Western blot analysis and 15% SDS-PAGE of purified recombinant proteins using Ni-NTA column chromatography: (**a** left) Electrophoretic mobility of purified Adnectin C. Lane 1: protein marker, lanes 2, 3: elution 1 and 2 of the purified protein (0.2 mg/ml of purified protein respectively); (**a** right) Electrophoretic mobility of purified Adnectin C-PAS#1(200). Lane 1: protein marker; lanes 2–4: elution 2–4 of the purified protein (0.1, 0.3 and 1 mg/ml of purified protein respectively). Western blot analysis identified the specified bonds for: (**b** left) Adnectin C; (**b** right) Adnectin C-PAS#1(200). The arrows show the band of the expected size of products. Full-length blots and gels are presented in Supplementary Figs S2–S4.

Supplementary Fig. S1 is schematic representations of the expression cassettes design. Digestion of the recombinant pET26a (+) and pET28a (+) vectors using *Sph*I-*Xho*I and *Xba*I-*Xho*I restriction enzymes results at the 650 bp and 1000 bp bands, respectively, in 1% agarose gel electrophoresis is shown in Supplementary Fig. S1b. The bands at approximately 11 and 100 kDa were detected by 15% polyacrylamide gel electrophoresis and Western blot analysis and matched the Adnectin C and Adnectin C-PAS#1(200) proteins, respectively (Fig. 1a,b). The results showed a noticeable reduction (4-fold) in the electrophoretic mobility of the PASylated protein.

### Particle size and zeta potential measurements

**Figure 2:**
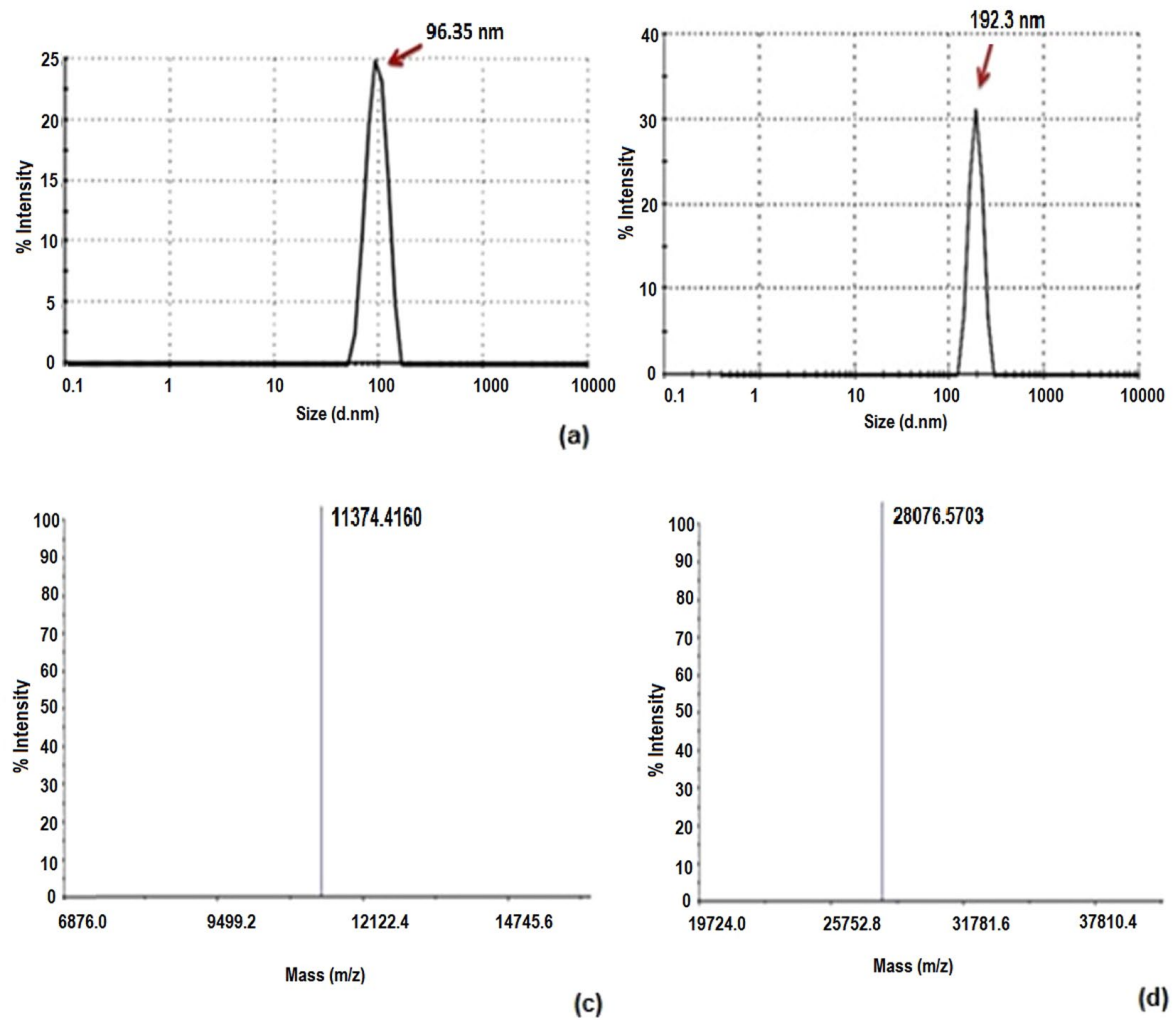
Hydrodynamic radius and mass spectrometric characterizations. Size distribution analysis of: (**a**) Adnectin C with main peak at 96.35 nm; (**b**) Adnectin C-PAS#1(200).with main peak at 192.3 nm. MALDI-TOF/TOF spectroscopy shows single peaks with a molecular weight of: (**c**) 11374.4160 Da for Adnectin C; (**d**) 28076.5703 Da for Adnectin C-PAS#1(200). Whole MALDI/TOF mass spectra are presented in Supplementary Fig. S5.

**Figure 3:**
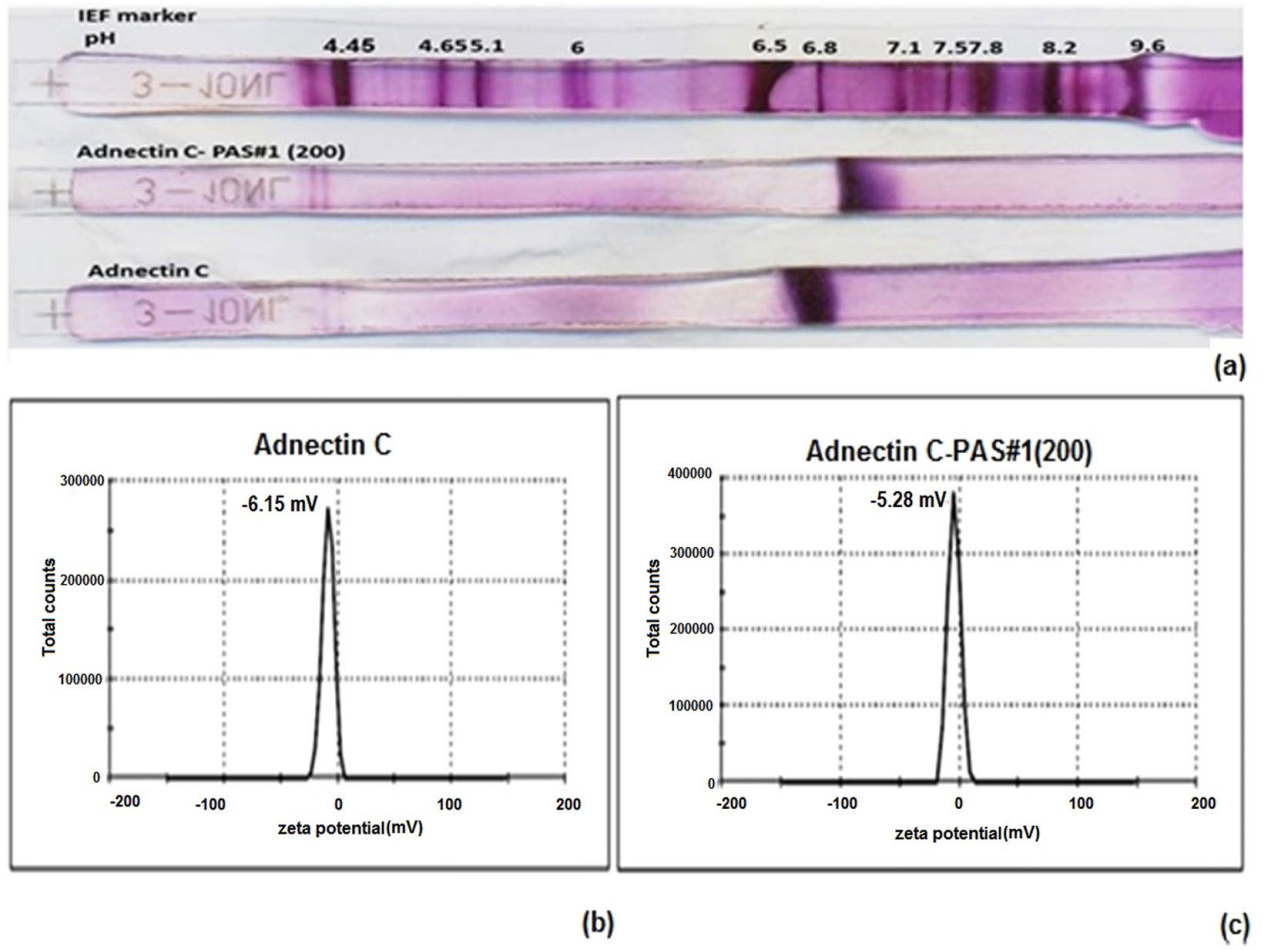
(**a**) IEF of PASylated Adnectin C and native protein on 7 cm IPG strips. Both proteins have similar net charges at pI = 6.5–6.8. Lane 1 is a marker, lane 2 is Adnectin C-PAS#1(200) and lane 3 is Adnectin C. Zeta potential distribution curves of: (**b**) Adnectin C; (**c**) Adnectin C-PAS#1(200). Measurements showed a negligible difference in zeta potential between Adnectin C-PAS#1(200) (−5.28 mV) and the native protein (−6.15 mV).

The effect of the PAS#1(200) sequence on the hydrodynamic radius and zeta potential of Adnectin C was investigated using DLS. Figure 2a,b show that PASylation resulted in about a 2-fold increase in the hydrodynamic radius. The zeta potentials were −6.15 and −5.28 mV for the native and PASylated samples, respectively, which reveals the negligible effect of the PAS#1(200) sequence on the surface charge of the native protein (Fig. 3b).

### MALDI-TOF mass spectrometry

Mass spectrometry data (11458.89 Da for Adnectin C and 28279.57 Da for Adnectin C-PAS#1(200)) exactly coordinated with the theoretical molecular mass of the proteins. The monodisperse composition was visible for both samples (just one peak) with no detectable shoulders or minor peaks related to impurities or truncated forms (Fig. 2c,d and Supplementary Fig. S5).

### IEF assay

The net charge of the PASylated protein was precisely characterized to study the effect of the PAS#1(200) sequence on the pI of the native protein. The data showed no obvious change in the pI of the native protein after attachment of the PAS sequence (pI = 6.5–6.8; Fig. 3a).

### Differential scanning calorimetry and thermogravimetry

**Figure 4:**
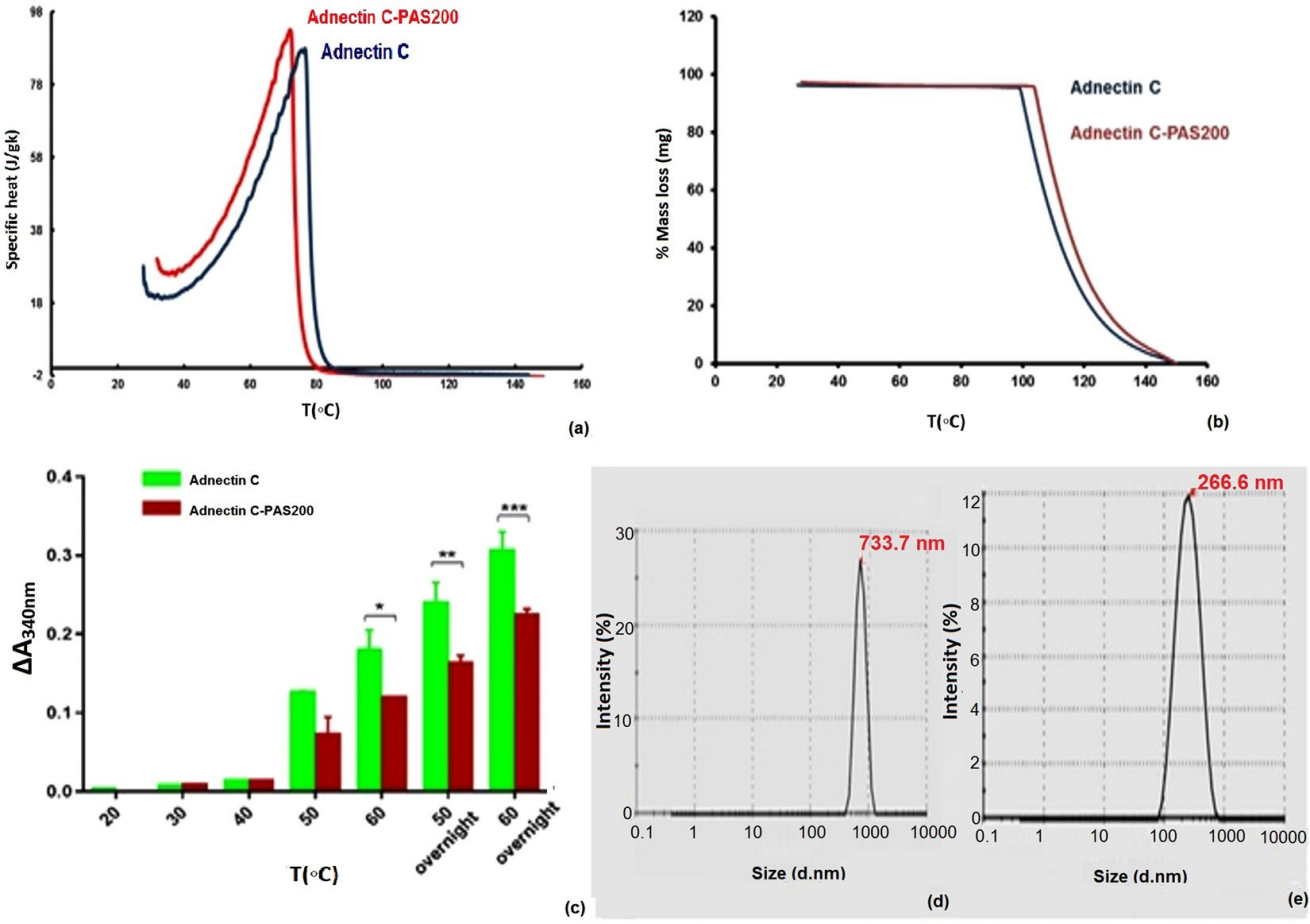
Thermo-analysis and protein aggregation assessment under different thermal and freeze-thaw conditions: (**a**) DSC thermograms; (**b**) TG curves of two-state unfolding PASylated Adnectin C and native protein. Samples were prepared in phosphate buffer at pH 6.8 and analyzed in inert atmosphere at a heating rate of 2 °C/min. A negligible shift in both thermograms was observed for PASylated Adnectin C relative to the native protein; (**c**) aggregation of PASylated Adnectin C and native protein after incubation at the defined temperatures and incubations times. A large increase in A_340nm_ after heating indicates lower thermal stability. Data are represented as mean ± SD (three replicates). Asterisks denote the significance levels (*p < 0.05, **p < 0.01, and ***p < 0.001); (**d**,**e**) DLS of freeze-thaw effect on protein aggregation of the recombinant proteins. Peak shifts were observed for: (**d**) the native (96.35 nm to 733.7 nm) and (**e**) PASylated protein (192.3 to 266.6 nm). Lower aggregated forms for PASylated were observed.

The effect of the PAS#1(200) sequence on the thermal behavior and stability of the native protein was studied using Differential Scanning Calorimetry (DSC) and Thermogravimetry (TG) techniques and the thermogram and thermogravimetric curves were plotted. Figure 4a and Table 1 shows that the denaturation temperature of the PASylated protein had a minor shift to the lower temperature side and showed similar thermal resistance in both samples. The ΔH_m_ (enthalpy of denaturation) of both samples was approximately equal, which represents a similar ordered secondary structure content. The ΔT_1/2_ value, which is the width of the calorimetric transition at half peak height, showed that PASylated and native proteins have similar cooperativity to unfolding. The thermograms showed only one peak with no detectable shoulders or minor peaks related to the thermal homogeneity of the samples. The nearly equal initial denaturation temperatures (IDT) of the PASylated and native forms of Adnectin C indicate that the PAS sequence had no noticeable effect on heat decomposition stability of the native protein (Fig. 4b).

**Table 1:** Thermal stability Characterization of the PASylated and native protein.

Protein	Denaturation Temperature (°C)	ΔT_1/2_ (°C)	Enthalpy of Denaturation (KJ)	Initial Decomposition Temperature (°C)
Adnectin C	75.3 ± 1.35	20.2 ± 0.42	2.036 ± 0.1′	100.675 ± 1.34
Adnectin C-PAS#1(200)	72.0 ± 0.21	20.0 ± 0.14	2.024 ± 0.09	104.10 ± 0.95

Data are represented as mean ± SD (three replicate).

### Thermal stability characterization of the PASylated and native proteins

To determine the effect of the PAS#1(200) sequence on changes in the aggregation propensity of the native protein, thermal and freeze-thaw assays were performed on the samples. Figure 4c shows that the A_340nm_ of both samples increased in a temperature-dependent manner. A large increase in A_340nm_ after heating likely relates to its lower thermal stability^10^. The data revealed that the PASylated protein was considerably more stable to aggregation at higher temperatures than the native protein. DLS analysis of the samples after freeze-thaw showed a 5.4-fold increase in the size of the native protein under the same conditions. This indicates that the PAS sequence had a stabilizing effect on the fused protein (Fig. 4d,e).

### Binding assay

**Figure 5:**
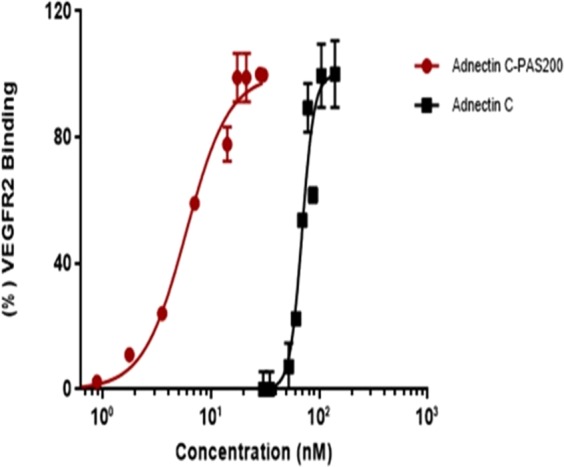
Binding assessment of Adnectin C and Adnectin C-PAS#1(200) to rhVEGFR2 by ELISA. Data are represented as mean ± SD (three replicates). PASylated Adnectin reached saturation at lower concentrations than the native protein.

The binding of PASylated Adnectin C and the native protein to rhVEGFR2 were studied using direct ELISA. Non-linear curve fitting shows EC_50_ values of 6.725 and 67.95 nM for Adnectin C-PAS#1(200) and Adnectin C, respectively. The results indicate that the attachment of the PAS sequence did not inhibit the binding ability of the native protein to the receptor (Fig. 5).

### Kinetic measurement by Surface Plasmon Resonance

Surface Plasmon Resonance (SPR) Studies on the thermodynamic affinity and binding kinetics versus rhVEGFR2 were performed by SPR spectroscopy on a CM5 chip. Table 2 shows the results of the kinetic and affinity parameters. The equilibrium dissociation constant (KD) of Adnectin C was greater than that for PASylated form. This data confirmed that PAS#1(200) polypeptide has improved the binding kinetics of the native protein.

**Table 2:** Calculated kinetic and affinity parameters for PASylated Adnectin C and native protein.

Protein	Ka^*^ (1/Ms)	Kd^**^ (1/s)	KD^***^ (M)
Adnectin C	9.868E + 4	5.643E − 5	5.718E − 10
Adnectin C-PAS#1(200)	1.560E + 5	2.476E − 5	1.587E − 10

^*^Association rate constant.

^**^Dissociation rate constant.

^***^Equilibrium dissociation constant.

### Toxicity assessment and Antiproliferative activity on HUVECs

**Figure 6:**
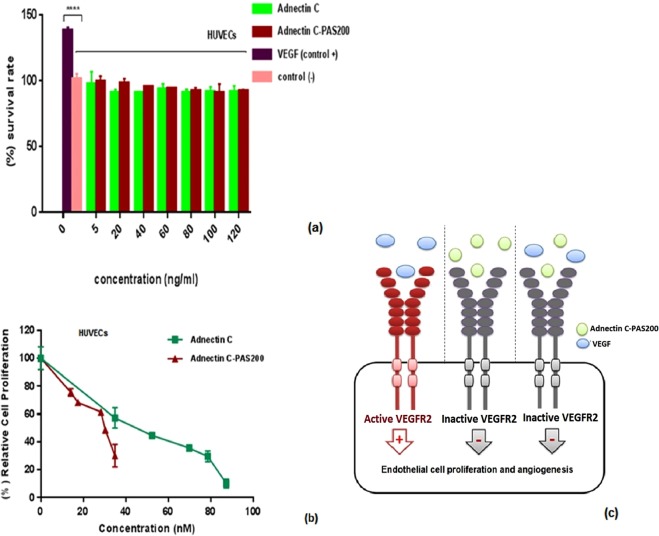
Toxicity assessment of Adnectin C, and Adnectin C-PAS#1(200) on HUVECs in culture (**a**), inhibition of VEGF-induced cell proliferation in HUVECs by recombinant proteins (**b**), and a schematic representation for mechanism of action of Adnectin C-PAS#1(200) (**c**). The data are represented as mean ± SD (three replicates). Asterisks show the significance of survival rate of samples versus VEGF group (****p < 0.0001).

MTT assay was employed to determine the toxic effects of recombinant proteins. The viability of human umbilical vein endothelial cells (HUVECs) after exposure to the different concentrations of recombinant proteins is shown in Fig. 6a. Both recombinant proteins were found to be nontoxic at 5–120 ng/ml, so this range was used for further *in vitro* experiments. Figure 6b shows the inhibitory effect of different concentrations of Adnectin C and Adnectin C-PAS#1(200) on HUVECs proliferation. Adnectin C and its PASylated form competitively inhibited HUVECs proliferation induced by activation of VEGFR-2 through VEGF-A in a dose-dependent manner. The differences in the anti-proliferative effect was statistically significant between the samples and untreated control HUVECs (p < 0.0001) and the samples and the VEGF group (p < 0.0001). The IC_50_ values for PASylated and native Adnectin C were 0.028 and 0.044 µM, respectively, which indicates that Adnectin C-PAS#1(200) was 1.57-fold more potent than the native protein for inhibiting the proliferation of HUVECs.

### Cell migration assay

**Figure 7:**
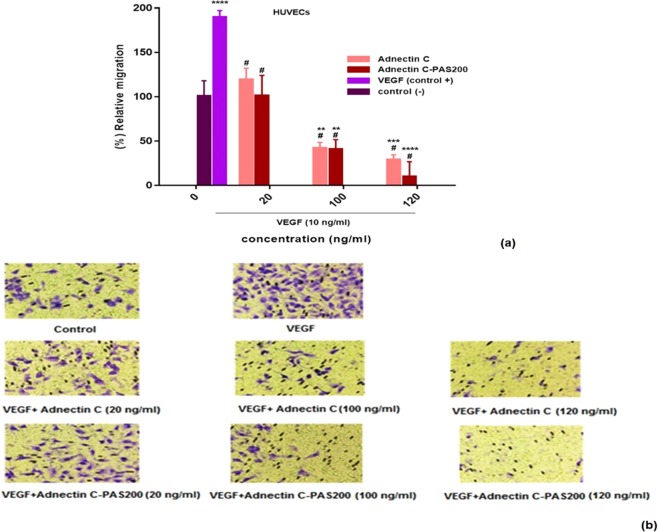
Adnectin C and Adnectin C-PAS#1(200) inhibited VEGF-induced migration of HUVECs: (**a**) inhibition by recombinant proteins on VEGF-induced migration of HUVECs through Transwell membranes. The data is represented as mean ± SD (three replicates); #denotes a significant difference from VEGF group (positive control) (p < 0.0001). Asterisks denote significant differences from the untreated (negative) control (**p < 0.01, ***p < 0.001, ****p < 0.0001); (**b**) representative photographs of stained membranes indicating both proteins considerably inhibited migration of HUVECs compared to the control (see original photographs in Supplementary Fig. S6).

Figure 7 shows the inhibitory effect of Adnectin C and Adnectin C-PAS#1(200) on the motility of HUVECs. HUVECs migrated through the Transwells membrane into the media motivated by the chemoattractant VEGF-A. Compared to the control (p < 0.0001), the VEGF-A induced migration of HUVECs was inhibited by both Adnectin C or Adnectin C-PAS#1(200) treatment in a dose-dependent manner. The maximum inhibition of endothelial cell migration was 87.27 and 34.90 nM (120 ng/ml) for Adnectin C and Adnectin C-PAS#1(200), respectively.

### Pharmacokinetic study

**Figure 8:**
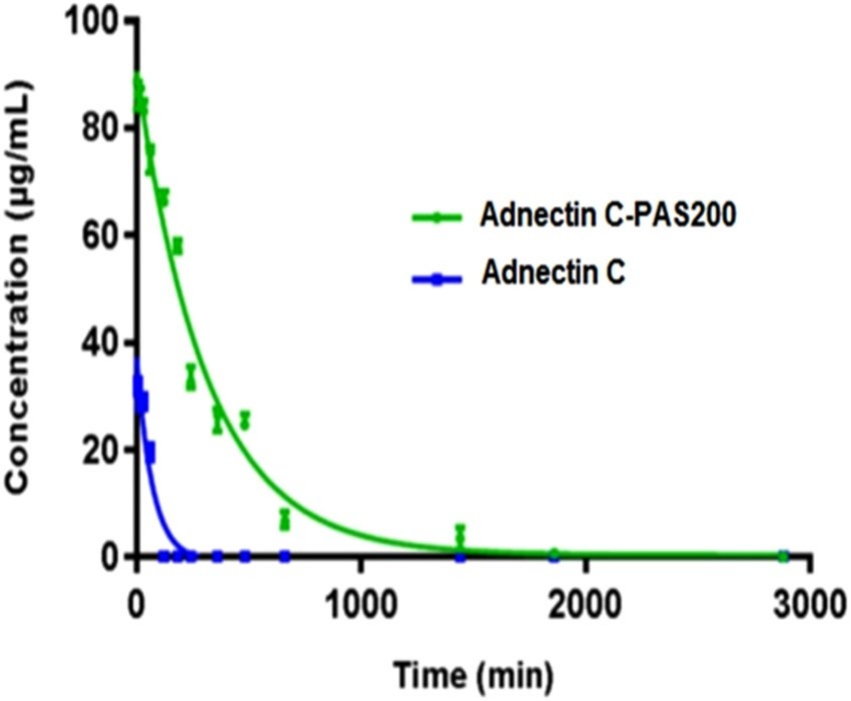
Pharmacokinetic profiles of Adnectin C and Adnectin C-PAS#1(200).after administration of 5 mg/kg to BALB/c mice. The graphs reveal the PASylated protein has a longer residence time in the circulation than the native protein.

The pharmacokinetic behavior of proteins was studied in female BALB/c mice. Adnectin C and PASylated Adnectin C are not detectable in the plasma after 1 and 48 h, respectively. The half-life of the terminal phase (T_1/2_) for PASylated Adnectin C was approximately 4.58-fold more than the native protein, which reveals that the PASylated protein has a longer residence time in the blood circulation. The other calculated pharmacokinetic parameters, such as the elimination rate constant and clearance, also decreased remarkably for the PASylated protein in comparison with Adnectin C (Fig. 8 and Table 3).

**Table 3:** Calculated pharmacokinetic parameters using a log-linear trapezoidal method for PASylated Adnectin C and the native protein.

Protein	T_1/2_ (min)	[AUC]_0–∞_ (µg.min/mL)	Elimination Rate Constant (h^−1^)	Clearance (mL/min)	PK factor
Adnectin C	49.41	1550.947	0.05	2.676889	1
Adnectin C-PAS#1(200)	226.2	30018.29	0.004	0.010707	4.578

## Discussion

A noticeable issue in the field of biopharmaceuticals is the short *in vivo* half-life of a substance, which requires its frequent administration^31^, and limits its efficacy^32^. This shortcoming often results from the molar mass of the bio-drug being below the renal threshold (40–60 kDa), which leads to rapid clearance from the circulation through glomerular filtration^33,34^. This constraint has eventuated a large body of work to develop strategies which minimize kidney filtration and improve the pharmacokinetic properties of such recombinant bio-drugs. PEGylation is an approved strategy to prolong the half-life of bio-drugs by increasing their hydrodynamic volume^17^.

Recently, recombinant approaches like the genetic fusion of peptide chains to PEG mimetics have been established. The basic concept of this genetic engineering is the replacement of PEG polymer with biodegradable, flexible and hydrophilic peptide chains which eliminate the need for chemical coupling and additional purification steps^21^. The current study aimed to extend the half-life of the anti-angiogenic agent Adnectin C using PASylation. The electrophoretic mobility, intact mass, receptor binding, kinetic parameters, size and charge characterization, toxicity, *in vitro* biological activity and pharmacokinetic parameters were studied. Additionally, the effect of the PAS sequence on the thermal stability and solubility of a PAS-fused protein were investigated for the first time.

In the structure-function relationship of Adnectins, besides diversified loops, residues from the N-terminus and/or the β strands are involved in the interaction with the target receptors or ligands^9^. The C-terminus PASylation strategy was employed to design a PASylated form of protein. It has been proposed that the PAS polypeptide will adopt a stable random coil in biological fluids that increases the hydrodynamic volume of the protein due to its unique structural conformation and physicochemical characteristics^21,26,35^. The results of assays indicate that the PAS sequence influences the physicochemical properties of the fused protein. For example, the electrophoretic mobility of the PASylated protein declined by a factor of 4.

It is known that the driving forces of proteins through a polyacrylamide network are size and charge^36,37^. The summation of the intact mass of the PAS#1 (200) sequence is about 17 kDa; thus, the PASylated protein must have a band at around 28.28 kDa, which is in accordance with the mass spectrometry results. The IEF results and those of other studies^21,35^ indicate that the PAS sequence does not alter the net charge of its fused partner. So, the reduction of electrophoretic mobility could be attributed to an increase in the surface hydrophilicity of the protein through the attachment of a hydrophilic, uncharged sequence of PAS. Because highly hydrophilic proteins bind less to the surfactant in one-dimensional SDS gel electrophoresis, slower migration has been reported for these proteins through the gel network. Moreover, the protein shape can affect gel migration. As demonstrated, the addition of a hydrophilic, random coil structure of PAS to the protein increased its hydrodynamic volume and substantially reduced its electrophoretic mobility^35^.

Stability is an essential factor which must be considered in biopharmaceutical developments^38^. DSC is popularly used for measuring the thermodynamic properties of proteins and DSC outputs (T_m_, ΔT_1/2_, and ΔH_m_) calculated from the peak of the transition temperature, width of the calorimetric transition at half peak height and area under the transition peak. These have been shown to be correlated with the thermal stability, cooperativity of protein unfolding and the extent of the ordered structure of a protein, respectively^13,39,40^.

Thermograms obtained from the DSC analysis of samples showed that the content of the ordered structure of the native protein did not change after fusion to the PAS#1(200) sequence. It has been confirmed in recent studies that the PAS#1(200) sequence does not alter the secondary structure of the fused biomolecules that is often essential for the stability and function of the protein^13,21,35^. Because the random coil structure of PAS#1(200) sequence lacks internal hydrogen bonding or hydrophobic interactions, equal amounts of thermal energy are needed to break the bond between the amino acid chains in the native and PASylated forms. Both proteins showed a similar unfolding transition pattern with unchanged cooperativity of unfolding. The denaturation and initial decomposition temperatures of PASylated and native protein were approximately equal, which shows the negligible effect of the PAS#1(200) sequence on the thermal stability of Adnectin C. These observations are similar to the effect of the PAS#1 sequence with a length of 600 amino acids on the melting temperature of human interferon α2b and human growth hormone^21^.

The zeta potential is a measure of the magnitude of the electrostatic interactions between charged surfaces^41,42^ and is another beneficial method of assessing the stability of biomolecules. It provides a detailed image of the colloidal stability of protein-containing systems^41,43^. For small molecules, a high zeta potential indicates greater stability for the molecule in the system from solvation or dispersion, which prevents subsequent aggregation^44^. The zeta potentials for PASylated and native Adnectin C were small and slightly negative with very similar values.

The aggregation that was observed during the short storage time or in the freeze-thaw assay of both samples under such thermodynamic conditions could occur because the attractive forces surpass electrostatic repulsion between adjacent, similarly charged particles in dispersion, which destabilizes the system and leads to aggregation of the dispersed particles^45^. This instability was more palpable in the shifts observed in the size distribution curves after samples were freeze-thawed which showed the growth of aggregated forms in both samples. The highlight of this was the increase of heat-induced aggregated forms observed for the native protein in comparison with the PASylated one (5.4-fold instability). It appears that the fusion of the PAS coding sequence made the protein more hydrophilic. This was demonstrated by the exposure of polar groups of unstructured chains of PAS sequences that are in contact with the solvent molecules^21^.

The increase in hydrophilicity of Adnectin C-PAS#1(200) and the solubilizing effect of the PAS sequence were confirmed by the linear relationship between temperature change and A_340nm_ because, in the absorbing light at a wavelength of 340 nm, the aggregated forms, not the soluble forms, absorb light^10^. Both proteins aggregated at temperatures below denaturation; however, the PASylated protein showed more resistance to heat-induced aggregation. This solubilizing effect is an attractive outcome of PAS sequence fusion and has been observed in XTENylation, another PEG mimetic peptide fusion technology^46^. Nonetheless, the anionic charge of the XTEN sequence can change the pI of its fusion partner^47^, reducing the receptor affinity and causing inappropriate tissue distribution in the body.

It was found that fusion of proteins with long polyglycine sequences have lower renal clearance, but show the propensity to produce aggregated forms^14,21^. Protein aggregation is the major concern during the development, manufacture, storage and shipping of biopharmaceuticals. Moreover, aggregates are noteworthy causes of drug immunogenicity, as well as being a drawback to efficacy and a safety risk^20,48^. PASylation is a promising technology that could eliminate the tendency toward aggregation of the PEG mimetic peptides without the introduction of charged side chains or additional downstream manipulation.

*In vitro* bioassays showed a substantial improvement in the biological activity of PAS fused protein. The curve for binding assay showed that the EC_50_ of PASylated protein had approximately one log shift to the lower concentrations compared to the native protein. This shift illustrates that despite PEGylated Adncetin C (CT-322), the affinity of Adnectin C to its receptor is upgraded after fusion to the PAS#1(200) sequence. Investigation of the binding kinetics indicated that PASylated Adnectin C binds to hVEGFR2 more effectively than the native protein, which improves the binding constant (KD). The data confirms the results of receptor binding analysis, which could be caused by the lack of the shielding effect of the PAS sequence on the protein which is commonly observed for PEG^5,20^, and has been reported for CT-322, and other PEG mimetic polypeptides^21^.

Previous studies on the mechanism of action of Adnectin C revealed that this recombinant protein blocks VEGF-induced receptor dimerization and the resultant receptor autophosphorylation. The blockade of VEGFR2 signaling also inhibits proliferation in HUVECs^4^. The anti-proliferative assay showed that the PAS-fused protein as well as native protein noticeably inhibited the proliferation of endothelial cells (ECs). Remarkably, the IC_50_ value of Adnectin C decreased after PASylation because the fusion protein was a more potent anti-proliferative than the native protein by a factor of 32. This effect was comparable with the commercially available PEGylated version of Adnectin C, in which the potency was reduced^4^. Regarding the binding assay result of PASylated Adnectin C and the knowledge on shielding effect of PEG polymer on the protein structure, the improved potency of PASylated protein is justifiable. The inhibitory effect observed in the anti-proliferative test could not arise from the cytotoxicity of the recombinant proteins because the range of concentration used for the test did not show any toxic effects on HUVECs without the addition of VEGF-A. Therefore, the fusion of the PAS#1(200) sequence to Adnectin C did not alter the mechanism of protein action.

It has been reported that anti-VEGFR2 antibodies which block VEGF binding sites, activate VEGFR2 partially^4^. The data from the current study indicates that the monomeric structure of Adnectin C and the PASylated form showed no such adverse effects. This interpretation is based on analysis of the survival rates of HUVECs upon exposure to the native and PASylated proteins at a range of 5–120 ng/ml. The results showed no substantial difference between the treated and untreated cells.

The Transwell assay was employed to investigate cell migration *in vitro*^49^. The anti-migration effect of PASylated Adnectin C was observed at lower concentrations, which reveals a synergistic effect of PAS#1(200) on inhibiting the migration of ECs. This stronger inhibition also occurred at nontoxic concentrations and can be attributed to the high binding affinity of PAS-fused protein to its receptor.

The balance between drug clearance and extravasation (the movement of drug out of the blood vessel and into the tumor) is a critical aspect of drug delivery^50^. Schlapschy *et al*.^21^ demonstrated that PAS-fused proteins are resistant to serum proteases and the kidney plays the key role in their clearance from the body. Elimination is carried out by the kidney by means of filtration through pores with a size comparable to the hydrodynamic diameter of the protein. Proteins with hydrodynamic radii that are smaller than the glomerular pores pass easily from the pores of the glomerulus. Other parameters affecting renal filtration of biopharmaceuticals include their molecular weight, shape, molecular conformation and flexibility^50^. In order to achieve a substantive therapeutic efficacy, improvement in the pharmacokinetics is inevitable for maintenance of the drug concentration in the therapeutic index without increasing the frequency of administration.

Bristol-Myers Squibb recently developed serum albumin binding Adnectin (PKE Adnectin) to compensate for the poor pharmacokinetics of Adnectin; however, this form shows low nanomolar affinity for human serum albumin and significant human and non-human primate pharmacokinetic parameters when compared with the non-binding form. MOA studies indicate that PKE Adnectin does not undergo FcRn-mediated recycling. Thus the long *in vivo* half-life of the PKE Adnectin can likely be attributed to the large size of the fusion and the reduction in renal clearance^51–53^. A PCSK9 Adnectin-Fc fusion protein is under development by this company that appears to be efficacious and has long-lasting effects on lowering low-density lipoprotein cholesterol in a cynomolgus monkey model^51,54^. The main disadvantages reported for Fc-fusion proteins are their expensive production because of the need for eukaryotic expression systems^55^ and development of an appropriate linker region for conjugating the effector molecule to the Fc region^56^.

Pharmacokinetic studies in mice have shown an improvement in the pharmacokinetic profile of PAS-fused proteins that could be explained by an increase in the hydrodynamic volume of the protein. A remarkable increase in the terminal half-life by a factor of 4.58 of the native protein was observed after PASylation. Additionally, the clearance of Adnectin C-PAS#1(200) was approximately 157-fold less than of the native protein. This improvement in the pharmacokinetic profile was previously observed for PEGylated Adnectin^4^, PAS#1(200) fused erythropoietin^25^ and anti-VEGF-A nanobodies^35^. The half-life extension factor of PASylated Adnectin is more than for PEGylated Adnectin^4^, moreover, this strategy lacks of most of the limitations of PEGylation. The main rout of excretion of Adnectin from the body is glomerular filtration^57,58^. By applying strategies based on the increasing of molecular hydrodynamic volume like PASylation, this mechanism remains but acts in a slower mode. The cytotoxic effects may raise from such extension half-life due to residence of active drug (Adnectin C) in the body. There are no data published on the toxicity of PAS sequences in the human so far, although, *in vivo* studies^21^ have shown that these stable sequences have no toxicity. It is noteworthy that PEGylated Adnectin lacking severe toxicities at the maximum tolerated dose^59^, but, decision on the safety of Adnectin-C PAS#1(200) must be performed after clinical trial data.

## Conclusion

Engineered Adnectin C clearly reveals a boosting effect of PASylation in stability, pharmacokinetic parameters and biological activity, besides maintaining its pharmacological effects *in vitro*. However, Pre-clinical data is needed to confirm the superiority of PASylated Adnectin-C over Adnectin C. PASylation could be an appropriate approach for half-life extension of Adnectins with features comparable to PEGylation. On the basis of these results, PASylated Adnectin C may be a promising drug candidate for clinical therapy.

## Methods

### Reagents and media

Recombinant human VEGF-A and recombinant human VEGFR2 were obtained from R&D Systems (Minneapolis, MN). HRP-conjugated anti-His antibody was purchased from Sina Biotech (Tehran, Iran). Restriction enzymes, and DNA ladder were obtained from Fermentas (Thermo Fisher Scientific Inc., Waltham, MA). All other chemicals were procured from Merck (Darmstadt, Germany). HUVECs were obtained from Isfahan University of Medical Sciences (Department of Cardiovascular research center, Isfahan, Iran). Female BALB/c mice were purchased from Pasteur Institute of Iran. Animal study was approved by Animal Ethics Committee of Pasteur Institute of Iran (Ethical code: IR.PII.REC.1397.68) and the methods were carried out in accordance with the relevant guidelines.

### Expression cassettes design

Two distinct gene expression cassettes containing the C-terminal 6xHis-tagged Adnectin C coding sequence (SEQ ID NO: 4)^60^, with or without the PAS#1(200) sequence [ASPAAPAPASPAAPAPSAPA] were designed, synthesized, and sub-cloned into the pET28a (+) and pET26b (+) vectors, respectively using *Nde*I and *EcoR*I restriction sites. Codon optimization was done for the high expression of proteins in *E. coli* strains, and restriction digestions were performed for confirmation of constructs.

### Protein expression and purification

Recombinant expression vectors were transformed into *E. coli* BL21 (DE3) pLysS according to the standard protocol12. The cultured colonies were then induced with 1 mM IPTG, shaken for 12–18 h at 25 °C and the cells harvested by centrifugation at 6000 g for 15 min. The expressions were analyzed on 12% SDS-polyacrylamide gels using Coomassie brilliant blue R250 (Sigma-Aldrich, St. Louis, MO) staining method (Mini-PROTEAN Tetra cell^©^, Bio-Rad, USA). Identification of the expressed proteins was performed by western blotting using Mini Trans-Blot® system (Bio-Rad, USA). Briefly, the protein bands were transferred (135 mA, 1.5 h) to a nitrocellulose paper and non-specific binding was blocked with blocking buffer (2.5% skim milk in PBS). Subsequently, HRP-conjugated Anti-His monoclonal antibody (1:4000 dilution, Sina Biotech, Iran) was added and incubated at room temperature for 2 h. After three washes (each 5 min) with 2.5% skim milk in PBS/T (PBS containing 0.1% Tween 20), Adnectin C and Adnectin C-PAS#1(200) protein bands were revealed with DAB staining (Sigma-Aldrich, USA).

For purification, the cells were resuspended in lysis buffer (NaH_2_PO_4_ 100 mM, Tris-Cl 10 mM, urea 8 M, adjusted to pH 8), sonicated on ice bath at 5 W for four 60 sec pulses separated by 10 sec intervals, centrifuged at 6000 g for 45 min, and the supernatant was filtered and loaded onto a HisTrap column (Amersham Biosciences, Piscataway, NJ). Tagged proteins were eluted using a buffer containing 50 mM NaH_2_PO_4_, 500 mM NaCl and 250 mM imidazole adjusted to pH 8, dialyzed against PBS (pH 7.2), and concentrated by ultrafiltration using Amicon Ultra centrifugal filter units (3000 MWCO; 15 ml; Millipore, Billerica, MA). The concentration of purified proteins was measured using Picodrop Microliter UV/Vis Spectrophotometer (Picodrop Ltd., Hinxton, UK) at a wavelength of 280 nm and total proteins concentrations were measured using the Bradford method^61^. For endotoxin removal of the protein solutions, a phase separation technique was employed using Triton X-100. The samples were treated with 0.1% Triton X-100 at 4 °C for 0.5 h. The samples were warmed to 37 °C for 15 min whereupon two phases formed. The Triton X-100 phase containing the endotoxin was separated by centrifugation (10 min, 10000 g). This process was repeated three times and the endotoxin content of samples was finally quantified using LAL QCL 1000-TM kit (LONZA, USA).

### Particles size and zeta potential measurements

Adnectin C and Adnectin C -PAS#1(200) proteins were assessed for their Zeta potential and mean particle size using ZetaSizer (Nano ZS, Malvern Instruments, UK) at a concentration of 0.5 mg/mL in PBS buffer. The measurements were accomplished at a wavelength of 633 nm, in which temperature was kept at 25 °C. Particle size distributions were calculated by the average of six measurements. Zeta potential was estimated on the basis of electrophoretic mobility under an electric field, as an average of 30 measurements.

### MALDI-TOF mass spectrometry

Matrix-assisted laser desorption/ ionization (MALDI) mass spectrometry was used to analyze the intact mass of the samples. Before MALDI-TOF analysis, desalting of solutions was performed by passing through C18 Zip-Tip reverse phase chromatography pipette tip (Millipore, Bedford, USA) according to the manufacturer’s instructions. The samples were then spotted on MALDI plate mixed with an equal volume of matrix solution of sinapinic acid in 50% ACN containing 0.1% TFA, air dried, and analyzed with a MALDI-TOF/TOF mass spectrometer (Applied Biosystems 4800 MALDI TOF/TOF), operated in high linear positive mode. Finally, the data were interpreted and processed using Data Explorer software version 4.0 (Applied Biosystems, USA).

### IEF assay

Thirty micrograms of each purified recombinant protein sample was separately applied to immobilized pH gradient (IPG) gel strips (pH 3–10 NL, 7 cm; BioRad, USA) in a total volume of 125 µl of rehydration solution (7 M urea, 2 M thiourea, 4% CHAPS, 2% carrier ampholytes, 50 mM dithiotreitol and 0.001% bromophenol blue). IEF standard (pI 4.45–9.6, Bio-Rad, USA) was applied for pI calibration. After rehydration for 16 h, protein separation was carried out by IEF at 20 °C using 50 µA/strip for 14,000 Vh at the maximum of 4,000 V in the Protean IEF cell (Bio-Rad, USA). After incubation in IEF gel staining solution (Bio-Rad, USA), the gels were de-stained with de-staining solution (20% ethanol, 5% acetic acid), and finally dried and scanned.

### Differential Scanning Calorimetry and Thermogravimetry

DSC and TG experiments were performed using a STA PT1600 thermo-analytical instrument (Linseis, USA). DSC analysis was implemented on each sample at a concentration of 1.0 mg/ml, and in the TG experiment, 2 mg of each freeze-dried protein was used. The scanning rate was 2 °C/min from 25–150 °C under nitrogen atmosphere. A filtering period of 5 sec was selected to reduce baseline noise for all samples.

### Freeze-thaw stability

Freeze-thaw testing was conducted by exposing the recombinant proteins to freezing temperature (−20 °C) for one month. The samples were then placed at room temperature for 24 h. The aggregation forms were analyzed based on the size changes of samples using ZetaSizer (Nano ZS, Malvern Instruments, UK).

### Thermal stability screening

The method was described previously [12]. Briefly, the purified proteins were dialyzed against phosphate buffer saline (PBS) and filtered through 0.22 µm syringe filters. The concentration of each sample was adjusted to 0.10 mg/ml with PBS, then 200 µl of each sample was dispensed into a 96-well, flat-bottomed plate. To stop evaporation, the samples were covered with 70 µl of mineral oil per well. The plate was incubated at 20, 30, 40, 50, and 60 °C for 2 h. Also, one plate was incubated at 60 °C for 24 h. The absorbance was read at a wavelength of 340 nm before and after each incubation using a microtiter plate reader (Epoch; BioTek Inc., Winooski, VT, USA). After the incubations, an increase in the absorbance was contemplated as a measure of heat-induced aggregation.

### Binding assay

The microtiter plates (96 well, NUNC, Roskilde, Denmark) were coated with 100 µl of the recombinant hVEGFR2 (R&D Systems, USA) at a concentration of 5 µg/mL in PBS (pH 7.4) overnight at 4 °C. After washing the wells with PBS containing 0.1% Tween 20 (PBS/T), blocking step was carried out using 200 ml 2.5% (w/v) bovine serum albumin for 2 h, and the wells were washed three times with PBS/T. To study the binding ability of Adnectin C and its PASylated form to VEGFR2, various dilutions of the recombinant proteins were added to microtiter plates in the same buffer for 2 h. The wells were further washed three times with PBS/T and incubated for 2 h with the anti-His HRP-conjugated monoclonal antibody (1:4000 dilution in PBS/T, Sina Biotech, Iran). After washing four times with PBS/T, 100 µl/well TMB (ready to use) (Pishtazteb, Iran) was added to detect enzymatic activity. After 15 min at 25 °C,the absorbance at 450 nm wavelength was measured using a microtiter plate reader (Epoch; BioTek Inc., Winooski, VT, USA).

### Surface plasmon resonance-based kinetics measurement

The binding affinity of Adnectin C and Adnectin C -PAS#1(200) for VEGFR2 was determined by surface plasmon resonance (SPR) biosensor. SPR experiments were accomplished by Biacore X100 (GE Healthcare) at 25 °C using HBS-EP running buffer (10 mm HEPES pH 7.4, 150 mm NaCl, 3 mm EDTA, 0.005% (v/v) detergent p20, pH 7.4) on CM5 (CM5 carboxymethyl dextran) sensor chip (GE Healthcare).

Anti-His antibody (from His Capture Kit, GE Healthcare) was amine coupled in the active and reference flow cell of a CM5 chip, according to the manufacturer’s instructions. Briefly, the surface was activated by injecting a solution containing 0.2 M N-ethyl-N -dimethylaminopropylcarbodiimide (EDC) and 50 mM Nhydroxysuccinimide (NHS) for 7 min. Anti-His antibody was diluted to 50 μg/mL in 10 mM Na-acetate, pH 4.5, and injected during 7 min, then the surface was blocked by injecting 1 M ethanolamine at pH 8.5 for 7 min. Immobilization levels in the range 6000–8000 RU were used. His-tagged native protein and PASylated Adnectin C (ligands) were injected at an appropriate concentration at flow rate of 5 μl/min for 180 s in active flow cell only, with a surface density of about 300 resonance units (RU). Multi cycle kinetics procedure was applied. A range of rhVEGFR2 (Genescript, Germany) concentrations (50, 25, 12.5, 6.25, and 3.125 µg/ml) was injected into flow cells, with a contact and dissociation time of 180 and 500 seconds, respectively. After each cycle, the chip was regenerated with regeneration buffer (10 mM glycine-HCl at pH 1.5) to eliminate all the binding proteins. Responses from an empty flow cell and from buffer injections were subtracted from recorded values. Kinetic analyses were performed by Biacore X100 evaluation software version 2 and affinity parameters were finally determined.

### *In vitro* cell toxicity assay

HUVECs were cultured according to the supplier’s directions. Briefly, HUVECs between passages 3 to 7, were dispensed into 96-well microplate (NUNC, Roskilde, Denmark) at a density of 2,000 cells/200 µL/well in DMEM containing 10% FBS and incubated for 24 h at 37 °C in a standard CO_2_ incubator. Then, various concentrations (0–120 ng/ml) of Adnectin C and Adnectin C-PAS#1(200) were added and the plates were incubated for a further 48 h. The media was replaced with 100 µl RPMI1640 and 10 µl MTT (12 mM) was added to the wells and incubated for 4 h at 37 °C. The cultures were then solubilized and the spectrophotometric absorbance was read at 570 nm wavelength using a microtiter plate reader (Epoch; BioTek Inc., Winooski, VT, USA). Finally the percentage of viable cells was calculated relative to the untreated controls.

### Antiproliferative assay

To assess the antiproliferative effect of proteins, the same toxicity procedure was performed with some modifications. Briefly, HUVECs, between 3 to 7 passages, were seeded at a density of 2,000 cells/well in 96-well flat-bottomed titer plate (NUNC, Roskilde, Denmark) and incubated for 24 h according to the supplier’s directions. Then, hVEGF165 (15 ng/mL, R&D, USA), and various concentrations of Adnectin C (0–90 nM), and Adnectin C-PAS#1(200) (0–40 nM) were added to each well. The cells were incubated for 48 h and the number of viable cells was determined by MTT assay kit (Bio-Idea, Iran) according to its manual instructions. Finally, cell proliferation was calculated relative to VEGF-A received groups.

### Cell migration assay

Serum-starved HUVECs were seeded at a density of 5 × 10^4^ cells per well, into the upper compartment of the corning Transwells (Sigma-Aldrich, USA). Defined concentrations (20, 100, and 120 ng/ml) of the Adnectin C or Adnectin C-PAS#1(200) were added to the upper chamber and the Transwells were inserted in a 6-well plate containing 1 mL of the DMEM. The bottom chamber was supplemented with 10 ng/mL hVEGF165 (R&D Systems, USA). After 24 h incubation, migrated cells were fixed with methanol and stained with 10% Giemsa stain and the cell membranes were de-stained with distilled water. Micrographs were taken from the membranes using a digital camera (Canon USA Inc., Lake Success, NY), finally, the migrated cells were counted using ImageJ version 1.44 software and cell migration was calculated relative to the untreated controls.

### Pharmacokinetic study

The animal experiment was approved by the Institutional Animal Care and Use Committee Female BALB/c mice (18–20 g) were randomly divided into three groups (n = 6). Adnectin C and Adnectin C-PAS#1(200) groups were each dosed intravenously at 5 mg protein/kg in PBS via the tail vein. The control group received PBS at an equal volume of test proteins for 20 g mice. Blood samples were taken after 5 min, 15 min, 30 min, 1 h, 2 h, 3 h, 4 h, 6 h, 8 h, 12 h, 24 h, 36 h, 48 h, 72 h, and 96 h (group I: 15 min, 2 h, 6 h and 72 h; group II: 5 min, 4 h, 12 h, and 96 h; group III: 1 h, 3 h, and 24 h; group IV: 30 min, 8 h, and 36 h). The plasma was prepared by centrifugation at 4 °C and 14000 g for 20 min and stored at −20 °C. The concentrations of recombinant proteins in the plasma were determined using a homemade ELISA. Briefly, a 96-well microtiter plate (NUNC, Roskilde, Denmark) was coated overnight at 4 °C with 100 µl of recombinant hVEGFR2 (R&D Systems, USA) at a concentration of 5 µg/ml in PBS adjusted to pH 7.2 at room temperature. After that, the wells were blocked with 200 µl 2.5% (w/v) bovine serum albumin in PBS for 1 h and washed three times with PBS containing 0.1% Tween 20 (PBS/T). The plasma samples were prepared in dilution series in PBS/T supplemented with up to 0.5% (v/v) untreated mouse plasma were individually added to each well and incubated for 2 h at room temperature. The wells were washed four times with PBS/T and incubated for 1 h with 100 µl anti-His HRP-conjugated monoclonal antibody (1/3000 dilution in PBS/T, Sina Biotech, Iran). After washing four times with PBS/T, the enzymatic activity was measured by adding 100 µl TMB solution (ready to use) (Pishtazteb, Iran) to each well as chromogenic substrates. After 30 min at room temperature, the absorbance at 450 nm wavelength was measured using a microtiter plate reader (Epoch; BioTek Inc., Winooski, VT, USA). Purified recombinant proteins at defined concentrations were diluted in PBS containing 0.5% (v/v) untreated mouse plasma and used for drawing standard curves. Quantification of the recombinant proteins in plasma samples was performed by comparison with the standard curves. Finally, concentrations versus time were plotted using two decay regression model and the linear trapezoidal method was used for the calculating of area under curve (AUC) and pharmacokinetic parameters.

### Statistical analysis

All graph and curve fittings were performed using Prism version 6 and the level of significance was assumed less than 0.05. Student t-test and one way ANOVA statistical tests were used for comparing the level of significance between two and several groups in the experiments, respectively.

## Supplementary information

supplementary information

## Data Availability

All data generated or analysed during this study are included in this published article.
