# Supplementary material for: Enhancing bioactivity, physicochemical, and pharmacokinetic properties of a nano-sized, anti-VEGFR2 Adnectin, through PASylation technology
Source: Sci Rep. 2019 Feb 27;9:2978. doi: 10.1038/s41598-019-39776-0 (PMC6393559; doi:10.1038/s41598-019-39776-0)
Supplement: Supplementary file 1 — supplementary information [file 41598_2019_39776_MOESM1_ESM.docx]

**Supplementary information**

**Enhancing bioactivity, physicochemical, and pharmacokinetic properties of a nano-sized anti-VEGFR2 Adnectin, through PASylation technology**

Safieh Aghaabdollahian^1^, Reza Ahangari Cohan^1*^, Dariush Norouzian^1*^, Fatemeh Davami^2^, Mohammad Reza Asadi Karam^3^, Fatemeh Torkashvand^4^, Golnaz Vaseghi^5^, Reza Moazzami^2^, Sakineh Latif Dizaji^1^

^1^ Department of Nanobiotechnology, New Technologies Research Group, Pasteur Institute of Iran, Tehran, Iran

^2^ Biotechnology Research Center, Pasteur Institute of Iran, Tehran, Iran

^3^ Department of Molecular Biology, Pasteur Institute of Iran, Tehran, Iran

^4^ Department of Biotechnology, Pasteur Institute of Iran, Tehran, Iran

^5^ Isfahan Cardiovascular research center, Department of Pharmacology, Isfahan, Iran

Corresponding authors

*Reza Ahangari Cohan

Department of Nanobiotechnology, New Technologies Research Group, Pasteur Institute of Iran, Tehran, Iran

E-mail: cohan_r@yahoo.com

Postal Address: Pasteur Institute of Iran (IPI), No. 69, Pasteur Ave, Tehran, Iran

Tel.: +98-21-66403496

Fax+98-21-66480777

*Dariush Norouzian

Department of Nanobiotechnology, New Technologies Research Group, Pasteur Institute of Iran, Tehran, Iran

E-mail: dnsa@pasteur.ac.ir

Postal Address: Pasteur Institute of Iran (IPI), No. 69, Pasteur Ave, Tehran, Iran

Tel.: +98-21-66403496

Fax+98-21-66480777

**Supplementary Figures**

**
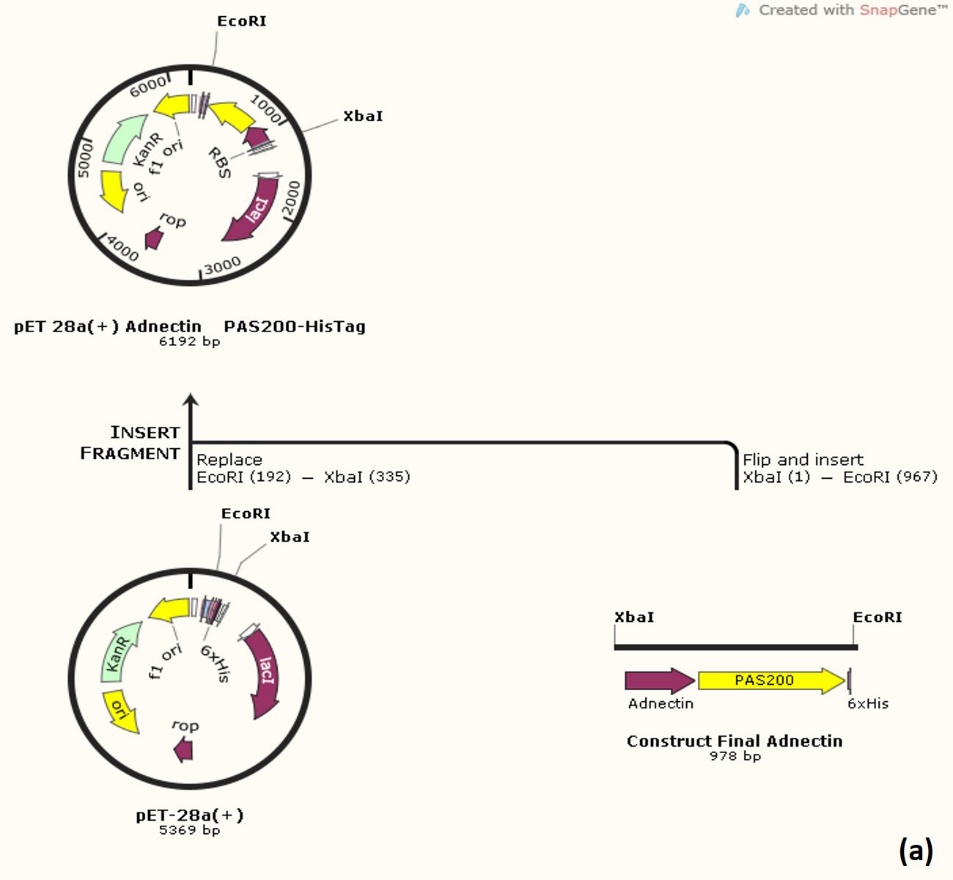
**


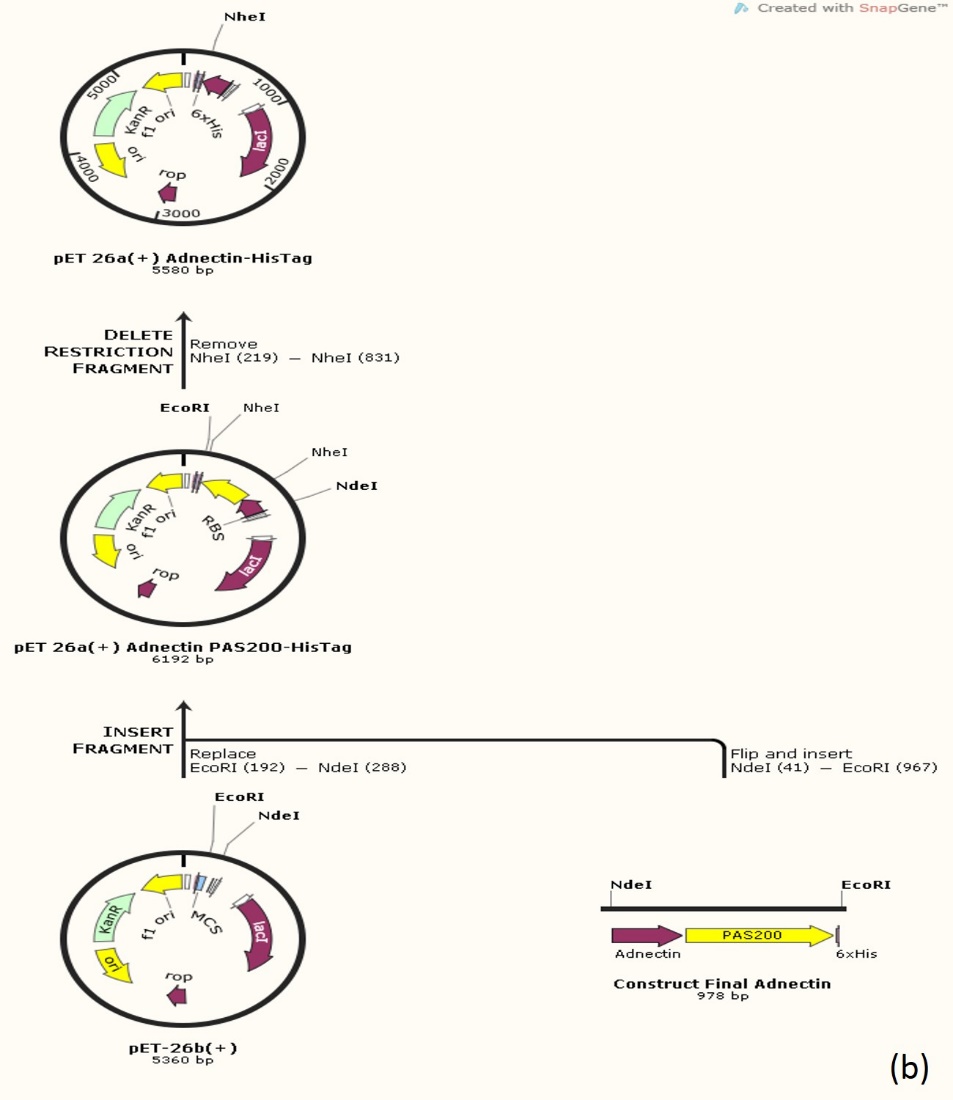

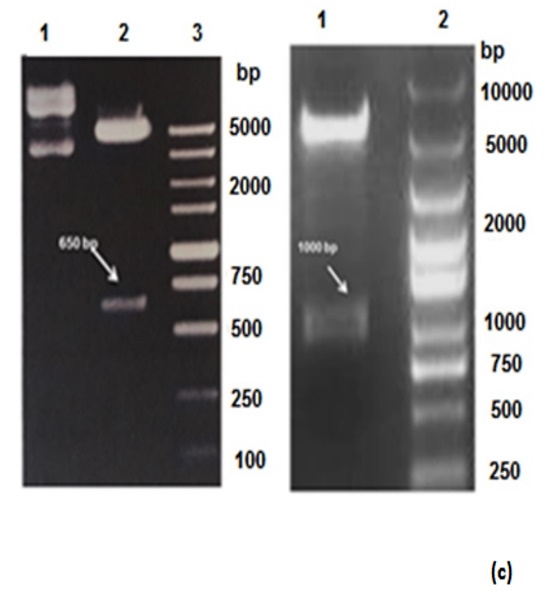


Supplementary Figure. S1. Schematic representation of the process of expression cassettes design are: (a) pET28a (+)/Adnectin C-PAS#1(200); (b) pET26b (+)/Adnectin C (the cassettes are designed using SnapGene software *v* 4.2); (c, left) gel electrophoresis of digested pET26a (+)/Adnectin C with *Sph*I-*Xho*I restriction enzymes. Lane 1 is the undigested pET26a (+)/Adnectin C expression vector, lane 2 is the digested pET26a (+)/Adnectin C and lane 3 is the DNA ladder; (c, right) gel electrophoresis of digested pET28a (+)/Adnectin C- PAS#1(200) with *Xba*I-*Xho*I restriction enzymes. Lane 1 is digested pET28a (+)/Adnectin C-PAS#1(200) and lane 2 is the DNA ladder.

**
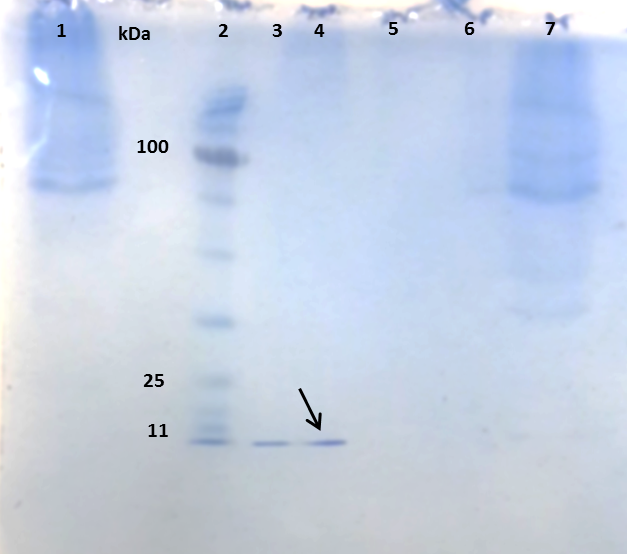
**

**Supplementary Figure. S2.** Electrophoretic mobility of recombinant Adnectin C on 15% SDS-polyacrylamide gel. Lane 1: un-induced sample; lane 2: protein marker; lane 3 and 4: elution 1 and 2 of the purified protein; lane 5-6: wash 1 and 2 of the purified protein. The arrows show the band of the expected size of product.

**
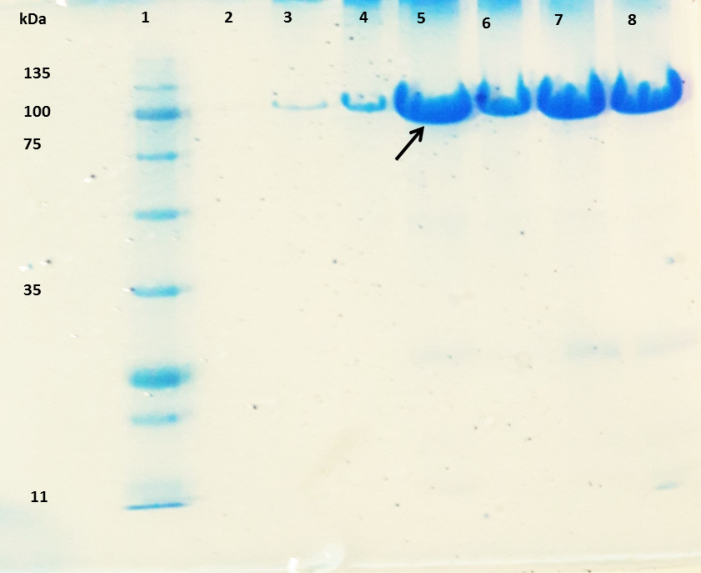
**

**Supplementary Figure. S3.** Electrophoretic mobility of Adnectin C-PAS#1(200) on 15% SDS-polyacrylamide gel. Lane 1: protein marker; lane 2: elution of un-induced sample; lanes 3-8: elution 1-8 of the purified protein. The arrows show the band of the expected size of product.

**
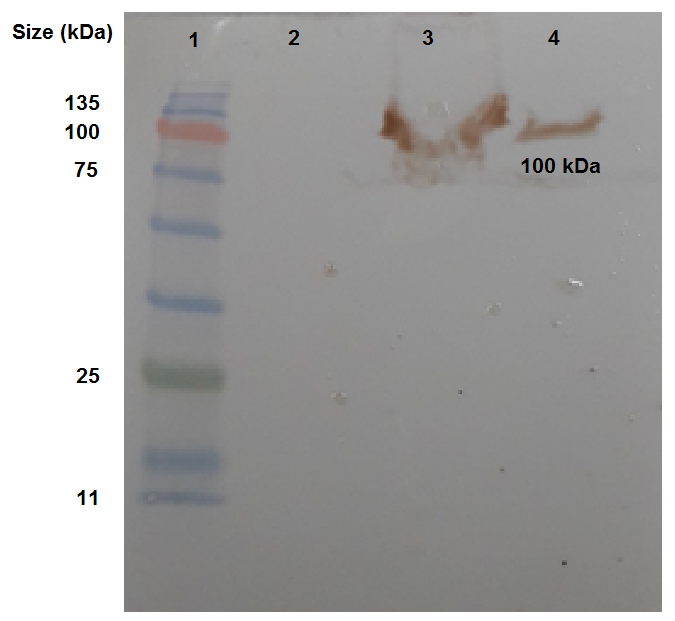
**

**Supplementary Figure. S4.** Western blot analysis of recombinant Adnectin C-PAS#1(200). Lane 1: protein marker; lane 2: un-induced sample. lane 3 and 4: induced samples with IPTG 1 M. Western blot analysis identified specified bonds for the PASylated protein.


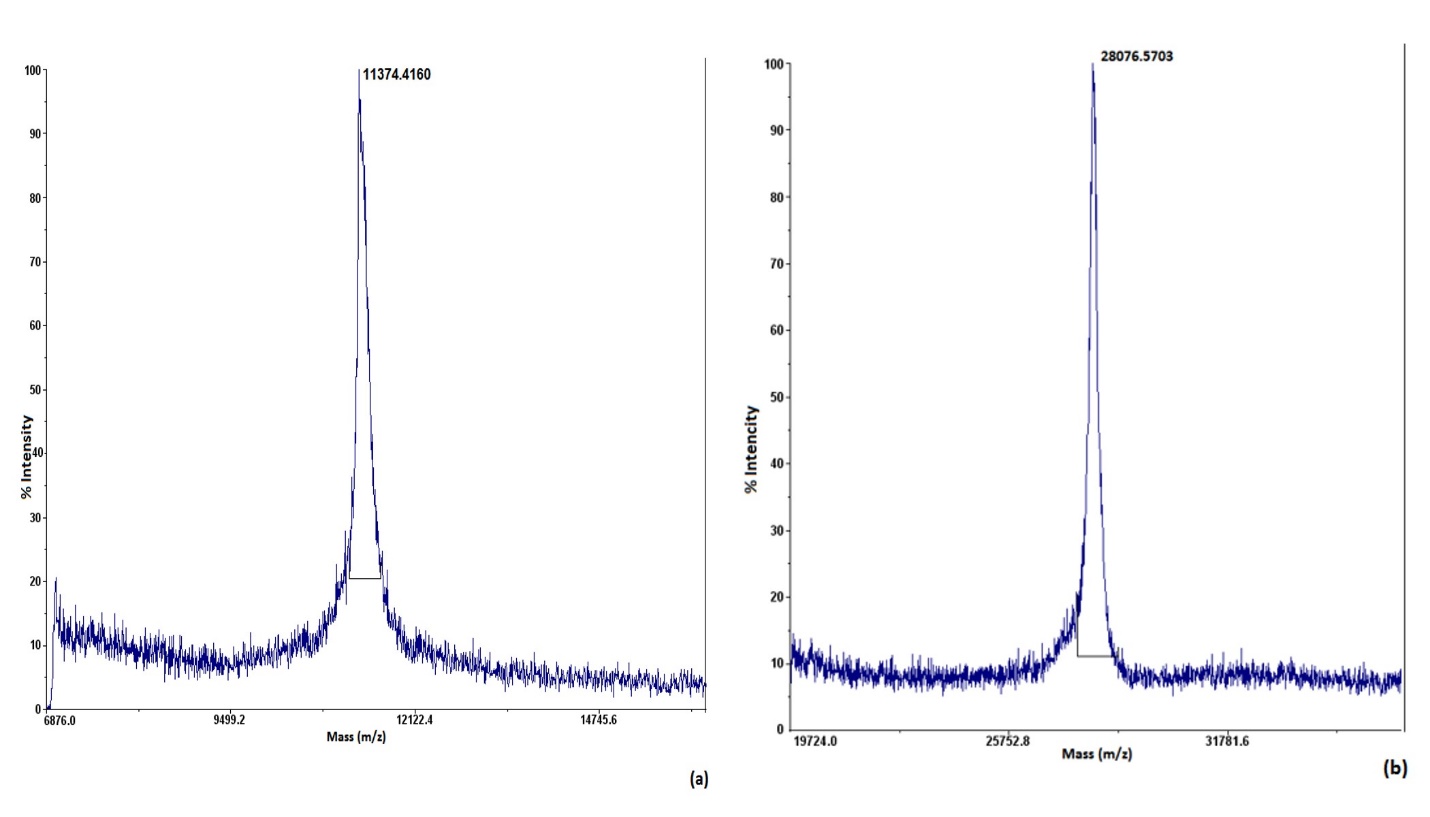


**Supplementary Figure. S5.** Mass spectrometric characterizations of PASylated Adnectin C and native form. MALDI-TOF/TOF spectroscopy shows single sharp peaks with a molecular weight of: (a) 11374.4160 Da for Adnectin C; (b) 28076.5703 Da for Adnectin C-PAS#1(200).

**
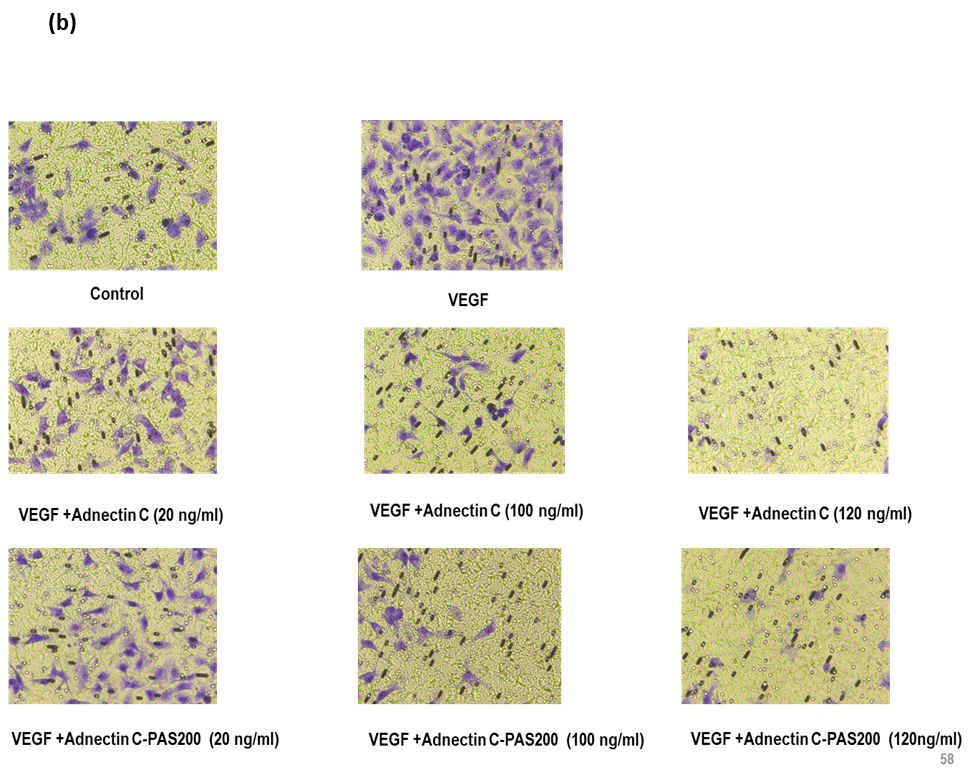
**

**Supplementary Figure. S6.** Adnectin C and Adnectin C-PAS#1(200) inhibited VEGF-induced migration of HUVECs: representative photographs of stained membranes indicating both proteins considerably inhibited migration of HUVECs compared to the control.
